# A Facile Method to Fabricate Anisotropic Extracellular Matrix with 3D Printing Topological Microfibers

**DOI:** 10.3390/ma12233944

**Published:** 2019-11-28

**Authors:** Zhen Gu, Zili Gao, Wenli Liu, Yongqiang Wen, Qi Gu

**Affiliations:** 1 School of Chemistry and Biological Engineering, University of Science and Technology Beijing, Beijing 100083, China; 2 CAS Key Laboratory of Bio-inspired Materials and Interfacial Science, Technical Institute of Physics and Chemistry, Chinese Academy of Sciences, Beijing 100190, China; 3 State Key Laboratory of Membrane Biology, Institute of Zoology, Chinese Academy of Sciences, Beijing 100101, China; 4 University of Chinese Academy of Sciences, Beijing 100049, China

**Keywords:** 3D printing, anisotropic extracellular matrix, microfibers, strength

## Abstract

Natural tissues and organs have different requirements regarding the mechanical characteristics of response. It is still a challenge to achieve biomaterials with anisotropic mechanical properties using an extracellular matrix with biological activity. We have improved the ductility and modulus of the gelatin matrix using 3D printed gelatin microfibers with different concentrations and topologies and, at the same, time achieved anisotropic mechanical properties. We successfully printed flat microfibers using partially cross-linked gelatin. We modified the 10% (*w*/*v*) gelatin matrix with microfibers consisting of a gelatin concentration of 14% (*w*/*v*), increasing the modulus to about three times and the elongation at break by 39% in parallel with the fiber direction. At the same time, it is found that the microfiber topology can effectively change the matrix ductility, and changing the modulus of the gelatin used in the microfiber can effectively change the matrix modulus. These findings provide a simple method for obtaining active biological materials that are closer to a physiological environment.

## 1. Introduction

Regenerative medicine research, such as tissue regeneration and repair, provides many conveniences for human life. The biological materials used in tissues and organs, such as ligaments, bones, cartilage and tendons, have different mechanical properties [1,2]. The development of biomaterials that meet the mechanical properties of human tissue has become an urgent issue in the field of tissue engineering. Hydrogels with a hydrating microenvironment are similar to biological tissues and suitable for providing anchoring, signaling and nutrient transport [3]. However, the preparation of the hydrogel usually involves the molecular assembly by polymerization or homogeneous dissolution in an aqueous medium, and the resulting hydrogel network is generally isotropic. To get closer to the functions and properties of natural tissues and organs, researchers need to develop anisotropic hydrogels to meet the cellular response to different mechanical properties [1,2,4]. The current methods commonly used by researchers to prepare anisotropic hydrogels include the introduction of 1D or 2D nanofillers in hydrogel matrices [5,6], orientation of the polymer network by external force [7] and introducing channel-like voids into the polymer [8]. In hydrogels that reproduce the in vivo environment, extracellular matrix-derived gelatin is non-immunogenic, naturally temperature sensitive, has a fixed cell recognition site and has been receiving increasingly more attention [9,10,11]. However, the mechanical properties of gelatin often do not match the biological tissue [11]. Biological tissues have not only a wide range of modulus ranges but also generally anisotropic mechanical properties that affect cell proliferation and differentiation [12]. Although the modulus can be increased by increasing the concentration of gelatin, the mechanical properties of gelatin are isotropic [13,14]. Looking for an easy way to achieve customized anisotropic gelatin using the bioactive gelatin itself remains a challenge. In order to realize the extracellular matrix customized by mechanical properties, we herein 3D printed gelatin filaments with amplitudes of 0 mm (AEM-0, anisotropic extracellular matrix referred to as AEM), 1 mm (AEM-1), and 2 mm (AEM-2), introduced the gelatin microfibers into the gelatin matrix, combined then with different gelatin concentrations, and obtained the gelatin material with customized mechanical properties. This results in an extracellular matrix with customized mechanical properties for the regeneration and repair of tissue organs.

## 2. Materials and Methods

### 2.1. Rheological Property Test

To determine the appropriate printing parameters, we analyzed the rheological properties of gelatin (Sigma, G-1890). The equipment was a rotating rheometer (MCR302, Anton Paar, Graz, Austria). The test mode was oscillation mode. The selected strain was 0.1%, the frequency was t 1 Hz, and a cone plate was used. The diameter of the tapered plate was 25 mm, with a taper of 2°. The spacing between the upper and lower cone plates was approximately 25 μm during the test. Different concentrations of gelatin samples were first incubated at 37 °C for 5 min before testing, and then subsequent experimental tests were initiated. The temperature-changing scanning method involved cooling from 37 to 4 °C, at a cooling rate of 1 °C/min. The mode of isothermal scanning was 2000 s under 4 °C.

### 2.2. 3D Printing Ink Preparation

According to the ratio of the mass of gelatin (Sigma, G-1890, St. Louis, MO, USA) to the volume of phosphate-buffered saline (PBS), 8%, 10%, 12%, and 14% (*w*/*v*) gelatin solutions were prepared and stirred at 60 °C for two hours. The homogeneous solutions were stored in a 4 °C refrigerator. 

### 2.3. Gelatin 3D Printing

The gelatin samples were taken from the refrigerator at concentrations of 8%, 10%, 12%, and 14% (*w*/*v*), placed at room temperature for about 20 min, and added to a 3D bio printer cartridge (Bio-Architects work station, Regenovo). The cartridge temperature was set to approximately 2 °C below the gel temperature. In order to smooth the printing wire, the temperature was finely adjusted according to different concentrations and the actual printing process. Prior to printing, the offset in the x and y directions was calibrated using an orthogonal optical micrometer. The diameter of the needle used for printing was 0.22 mm and the platform temperature was set to 4 °C. The gelatin microfibers were printed on the bottom of the dish according to the design graphic. We prepared 15 mM N-(3-dimethylaminopropyl)-N’-ethylcarbodiimide (EDC; Aladdin, E106172-25g)/6 mM N-hydroxysuccinimide (NHS; Aladdin, H109330-100g) in deionized water solution, and the gelatin samples were immersed in the solution for cross-linking for 1 h at 4 °C. The choice of EDC/NHS as a cross-linking agent is mainly due to two considerations. One is that there are carboxyl groups and primary amine groups in gelatin [15] and the formed amide bond, similar to a peptide bond, is beneficial for biocompatibility [16]. The other is that previous researchers have shown that biomaterials prepared by EDC/NHS as a cross-linking agent are biocompatible [15,16,17].

### 2.4. Gelatin Film Preparation

Samples were prepared according to 8%, 10%, 12%, and 14% (*w*/*v*) gelatin/PBS solution (*w*/*v*), and solutions were stirred at 60 °C for 2 h, and the pH of the PBS solution was in the range of 5.0–7.0. The mixed solution (12 mL) was added to the culture dish and then placed at 4 °C for 30 min, followed by addition of 1.5 mM EDC/0.6 mM NHS in deionized water, and cured at 4 °C for 1 h to prepare a 0.5 mm gelatin film.

### 2.5. Gelatin Mechanical Strength Test

The equipment used for the test was the ESM303 Force Test Stand (Mark-10, New York, NY, USA) with a 10 N capacity force gauge (M5-2). Samples were placed in PBS solution prior to testing to achieve a swelling balance and placed in a 25 °C water bath to achieve the same sample temperature test. The sample size was 30 mm × 10 mm × 0.5 mm using a scalpel. In order to better fix the sample with the clamp, the paper is used to increase the fixed area at both ends. The stretching speed is set to 20 mm/min. The Young’s modulus is calculated from the slope of the stress–strain curve in the 0.5%–5% region. Strain at peak stress is recorded as elongation at break. Three test samples were prepared for each sample.

## 3. Results and Discussions 

### 3.1. Design of Anisotropic Extracellular Matrix 3D Printed Topological Microfibers

**Figure 1:**
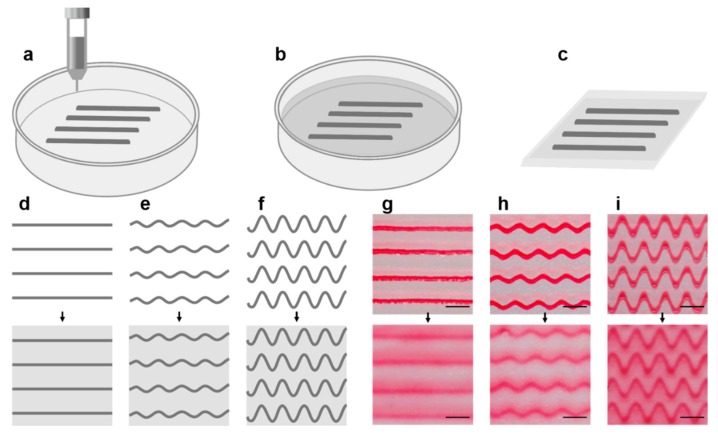
Preparation of anisotropic gelatin based on 3D printed gelatin microfibers. (**a**) 3D printed gelatin microfibers which are then cross-linked. (**b**) Pouring the gelatin solution and then cross-linking. (**c**) Obtaining an anisotropic gelatin based on microfibers. (**d**–**f**) Schematic diagram of microfiber and modified gelatin with different topological microfiber. The distance between the fibers is 2 mm, (**d**) shows a linear fiber. (**e**) A sinusoid with an amplitude of 1 mm and a period of 2 mm. (**f**) shows a sinusoid with an amplitude of 2 mm and a period of 2 mm. (**g**–**i**) The topographical curves corresponding to (**d**–**f**), respectively. The fiber is dyed in red for observation. In order to achieve better anisotropy and mechanical modification effects, the fibers and matrix were each used in different gelatin concentrations in subsequent experiments, which will be discussed in detail later. Scale bars: 2 mm.

The main design steps for the preparation of anisotropic gelatin from 3D printed gelatin microfibers are shown in Figure 1a–c (Video S1). First, the gelatin microfibers are printed in 3D, and then cross-linked with N-(3-dimethylaminopropyl)-N’-ethylcarbodiimide (EDC)/N-hydroxysuccinimide (NHS) [18]. Afterward, gelatin solution was added and also cross-linked. Anisotropic gelatin can be obtained. We optimized the gelatin matrix with straight lines and sinusoids of different amplitudes as shown in Figure 1d–f. We obtained three microfiber arrays with a spacing of 2 mm between the fibers and a period of 2 mm for both sinusoidal fibers with amplitudes of either 1 or 2 mm (Figure 1g–i). In order to use gelatin microfibers to regulate the mechanical properties of the gelatin matrix, the fiber and the matrix were obtained with different concentrations of gelatin solution. This will be discussed in detail in the following section of the manuscript.

### 3.2. 3D Printing Characterization of Gelatin

**Figure 2:**
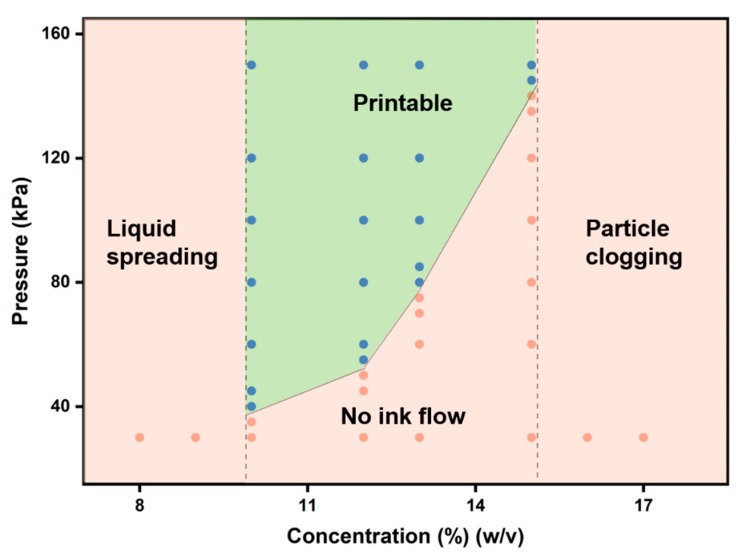
Printed phase diagram of gelatin solution. It shows 3D printed phase diagram of gelatin solution at 28 °C under different pressures and concentrations. The green area in the figure (the blue dot is the point that can be printed in experiment) is the printable area, and the orange area (the orange dot is the unprintable point found by the experiment) is the unprintable area.

**Figure 3:**
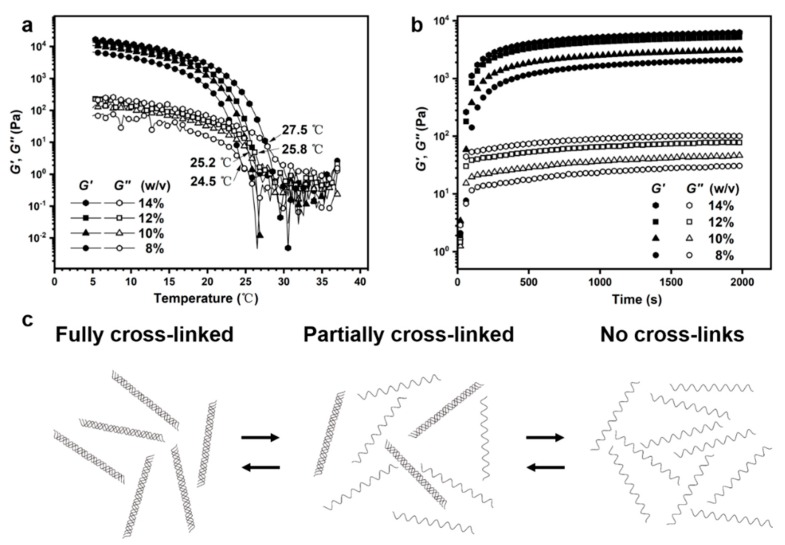
Gelatin rheological properties. (**a**) The storage modulus and loss modulus of different concentrations of gelatin as a function of temperature. The intersection of storage modulus and loss modulus is the gel temperature point. As the concentration increases, the gel point temperature increases. (**b**) The storage modulus and loss modulus of gelatin as a function of time at 4 °C. (**c**) Schematic diagram of the cross-linking state of the thermally responsive gelatin at different temperatures. When the temperature is much lower than the gel temperature, the gelatin is in a completely cross-linked state. When the temperature is further increased, the gelatin polypeptide chains gradually separate into a partially cross-linked state. When the temperature is above the gel temperature, the gelatin enters a solution state.

Gelatin has very good cell compatibility [19] and the temperature-sensitive mechanical properties which facilitate 3D printing directly through temperature control [20], and is widely used in regenerative medicine [21,22,23,24]. For subsequent 3D printing, we first explored the printing performance of gelatin solution at 28 °C under different pressures and concentrations (Figure 2). When the gelatin solution concentration is less than 10% (*w*/*v*), the gelatin solution flows out directly. When the concentration of the gelatin solution is greater than 15% (*w*/*v*), the gelatin is blocked in the tip of the nozzle and cannot be extruded. Between 10% and 15% (*w*/*v*), the gelatin solution can be extruded into fibers for 3D printing by applying the appropriate pressure. Also, the storage and loss modulus of gelatin samples (Figure 3a,b) increased with increasing concentration. Similar results were found in previous studies [20,25,26]: as the polymer concentration increased, the modulus of the sample increased. Thus, at lower gelatin concentrations (less than 10% (*w*/*v*)), the mechanical strength of the sample is too low to support the printed sample to maintain shape. At higher gelatin concentrations (greater than 15% (*w*/*v*)), the sample has high mechanical strength and smooth fibers cannot be extruded. It should also be noted that the 3D printing performance of gelatin solutions is also affected by temperature and will be discussed in detail in the next analysis. 

Gelatin is a thermally responsive bioactive material [27]. In order to achieve better printing, there are three main methods: direct printing of low-temperature gelatin [9]; streaming gelatin directly onto a low temperature stage [27]; and blending other polymers to increase gelatin viscosity [28]. In order to print a more accurate structure and maintain the composition of gelatin, we printed partially cross-linked gelatin which was possible due to the thermal response characteristics of gelatin [27]. As shown in Figure 3a, the storage modulus and loss modulus intersection point is the gel temperature point; different concentrations of gelatin have different gel temperatures. The corresponding gel temperatures of 8%, 10%, 12% and 14% (*w*/*v*) gelatin solutions are 24.5, 25.2, 25.8 and 27.5 °C respectively. This provides a reference temperature of printing for each concentration of gelatin solution. At about 2 °C below the gel temperature, gelatin is partially cross-linked (Figure 3c), the storage modulus of which is about two orders of magnitude lower than that of complete cross-linked gelatin gel. On the one hand, there is a certain modulus required to support the printed fibers though it is not too high nor difficult to extrude. With better printing characteristics [18], the printed fibers are relatively flat. We can use this ink to print fibers of different topologies (Figure 1). At the same time, at 20 °C, the storage modulus of gelatin increased with increasing concentration, from 497 Pa at 8% (*w*/*v*) to 2735 Pa at 14% (*w*/*v*). The fiber we printed was on a 4 °C stage. We tested the mechanical properties of gelatin over time at 4 °C (Figure 3b). It can be seen that the modulus of gelatin begins to increase faster, before the increase rate gradually diminishes. At 2000 s, the storage modulus of gelatin gel with 8%, 10%, 12% and 14% (*w*/*v*) reached 2114, 3087, 5180 and 6291 Pa, respectively.

### 3.3. Anisotropic Gelatin Based on Gelatin Modulus and Microfiber Topology

**Figure 4:**
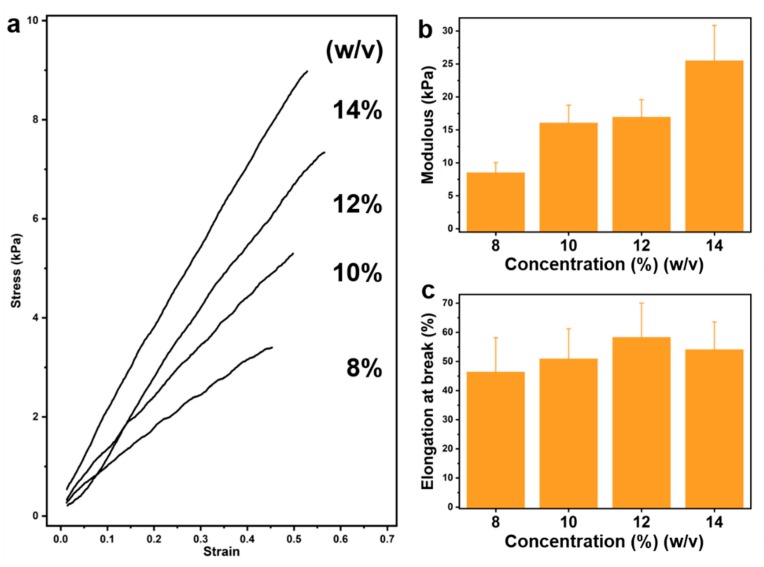
Stress–strain curves for cast samples without microfiber at different gelatin levels. As the concentration of gelatin increases, the modulus of the sample increases (**a**,**b**). The change in elongation at break is not very obvious (**c**).

We first analyzed the mechanical properties of the non-microfiber-filled gelatin at different concentrations (Figure 4). The higher the gelatin concentration, the greater the modulus, from 8.5 kPa of 8% (*w*/*v*) gelatin to 25.5 kPa of 14% (*w*/*v*) gelatin (Figure 4b), the modulus is increased by a factor of three times. Previous studies [14,20,26,29] and the rheological tests of gelatin (Figure 3a,b) also found that the modulus of the sample increased with increasing concentration. It is possible that the degree of cross-linking increases due to an increase in concentration [30]. At the same extent of gelatin concentration increase, the elongation at break of gelatin did not change significantly as the modulus changed. When the gelatin concentration increased from 8% to 14% (*w*/*v*), the elongation at break only increased by about 16%. This indicates that a certain range of bioactive materials with adjustable mechanical properties can be obtained by changing the concentration of gelatin. On the other hand, changes in the mechanical properties of gelatin are achieved by varying the concentration, which also affects the biological activity of the gelatin matrix [31] since an increase in the concentration of gelatin causes an increase in the modulus and the ligand points [25,32]. Next, we consider the introduction of microfibers that are also gelatin by 3D printing without changing the gelatin matrix concentration to optimize the mechanical properties of the gelatin matrix.

**Figure 5:**
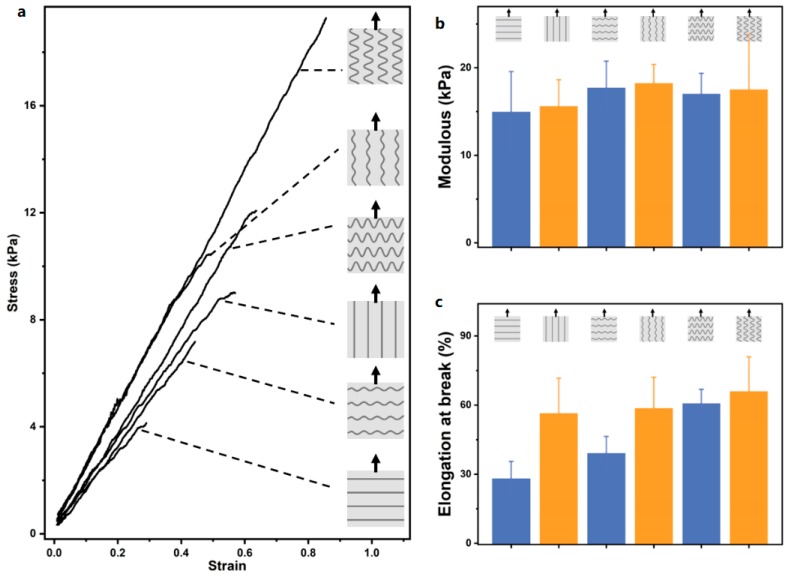
Modified gelatin matrix using different topological structures of gelatin microfibers. The experiment used 10% (*w*/*v*) gelatin microfiber to modify 8% (*w*/*v*) gelatin matrix. (**a**) The stress–strain curves of the gelatin matrix were obtained in both vertical and parallel directions to the microfibers. The gelatin matrix contains 3D printed gelatin filaments with amplitudes 0 mm (AEM-0), 1 mm (AEM-1), and 2 mm (AEM-2). (**b**,**c**) Modulus and elongation at break were measured in both the vertical and parallel directions to the microfibers of AEM-0, AEM-1, and AEM-2. The illustrations indicate the topology and direction of the microfibers in the sample.

**Figure 6:**
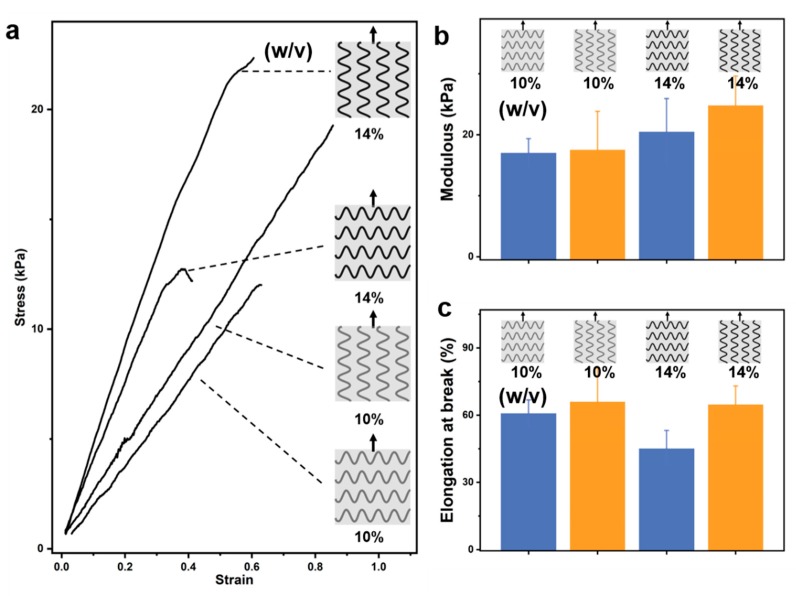
Mechanical properties of gelatin matrix modified by microfibers with different gelatin concentrations. (**a**) 3D printing 10% (*w*/*v*) and 14% (*w*/*v*) gelatin sinus microfibers modified 8% (*w*/*v*) gelatin matrix. The stress–strain curves of the samples along both vertical and parallel directions to the microfibers. Modulus and elongation at break (**b**,**c**) for different tensile directions of different samples obtained from the stress–strain curves.

We introduced 10% (*w*/*v*) gelatin microfibers into 8% (*w*/*v*) gelatin matrix. 3D printing makes it easy to obtain microfibers with different topologies. The introduction of topological microfibers can alter the stress–strain mechanical response of the gelatin matrix (Figure 5a). We found that the introduction of microfibers has a significant effect on the anisotropy of the elongation at break (Figure 5c). For example, AEM-0 has an elongation at break of 28.1% and 56.5% in both vertical and parallel directions, respectively. At the same time, compared with the 8% (*w*/*v*) gelatin matrix without microfiber, its elongation at break is 46.5%, and the elongation at break of AEM-2 increased obviously in both vertical and parallel directions, which were 60.1% and 66.0%, respectively. However, the microfibers of different topologies have little effect on the anisotropic modulus of the gelatin matrix (Figure 5b). We know that gelatin concentration has a great influence on the modulus. Therefore, we increased the gelatin concentration of microfibers to increase the modulus of the gelatin matrix. For the AEM-2 structure, we further increased the gelatin concentration of microfibers to 14% (*w*/*v*) (Figure 6). We found that the modulus of the gelatin matrix was significantly improved. The modulus reached 24.8 kPa (Figure 6b) in parallel with the fiber direction and the elongation at break also reached 64.8% (Figure 6c). We cultured cells for different times on the gelatin with elongations of 0% and 60%. As the culture time prolonged, the spreading area of the cells on the substrate with different elongation gradually increased. The spreading area was larger on the substrate with elongation 60% (Figure S1). This may be due to strain (60%) causing an increase in the modulus of the gelatin [33], which promotes cell spreading [34].

## 4. Conclusions

In conclusion, we used a 3D printing method to modify the gelatin matrix with gelatin microfibers of different concentrations and topologies. We found that with the thermal responsiveness of gelatin, partially cross-linked gelatin can be used to produce smoother microfibers for accurate printing. The increase in gelatin concentration can significantly increase the matrix modulus. When the gelatin concentration was increased from 8% (*w*/*v*) to 14% (*w*/*v*), the modulus was nearly three times that of the original. In order to improve the ductility of the matrix material and, at the same time, achieve anisotropy of the mechanical properties, a 3D printed gelatin microfiber array was introduced. When the gelatin concentration of the microfiber was 10% (*w*/*v*), compared with the microfiber-free 8% (*w*/*v*) gelatin matrix, the elongation at break of which is 46.5%, the elongation at break of AEM-2 increased significantly in both vertical and parallel directions, by 60.1% and 66.0%, respectively. This improved the ductility of the substrate. At the same time, AEM-0 had an elongation at break of 28.1% and 56.5% in both vertical and parallel directions, which improved the anisotropy of the matrix. Further, when the gelatin concentration of the microfibers is 14% (*w*/*v*), the modulus of the gelatin matrix is 24.8 kPa in parallel with the fiber direction, and the elongation at break was also 64.8%. By changing the gelatin concentration and topology of microfibers, we can easily adjust the mechanical properties and anisotropy of the gelatin matrix and provide a simple preparation method for obtaining bioactive materials with properties close to those of physiological conditions.

## Supplementary Materials

The following are available online at https://www.mdpi.com/1996-1944/12/23/3944/s1, Figure S1: Culture diagrams of cells at different times on substrates with elongations of 0% and 60%, Video S1: Fabricating anisotropic gelatin with 3D printing topological microfibers.
