# Supplementary material for: A Facile Method to Fabricate Anisotropic Extracellular Matrix with 3D Printing Topological Microfibers
Source: Materials (Basel). 2019 Nov 28;12(23):3944. doi: 10.3390/ma12233944 (PMC6926675; doi:10.3390/ma12233944)
Supplement: Supplementary file 1 [file materials-12-03944-s001.zip › supplementary/materials-643368-supple.docx]

Supplementary information of

A Facile Method to Fabricate Anisotropic Extracellular Matrix with 3D Printing Topological Microfibers

Zhen Gu ^1,2,†^, Zili Gao ^3,†^, Wenli Liu ^3^, Yongqiang Wen ^1^ and Qi Gu ^3,4,^*

^1^ School of Chemistry and Biological Engineering, University of Science and Technology Beijing, Beijing 100083, China; guzhen@ustb.edu.cn (Z.G.); wyq_wen@ustb.edu.cn (Y.W.)

^2^ CAS Key Laboratory of Bio-inspired Materials and Interfacial Science, Technical Institute of Physics and Chemistry, Chinese Academy of Sciences, Beijing, 100190, China

^3^ State Key Laboratory of Membrane Biology, Institute of Zoology, Chinese Academy of Sciences, Beijing 100101, China; gaozili18@mails.ucas.ac.cn (Z.G.); 13563533707@163.com (W.L.)

^4^ University of Chinese Academy of Sciences, Beijing 100049, China

***** Correspondence: qgu@ioz.ac.cn; Tel.: +86-10-64807315

^†^ Both authors contributed equally to this work.


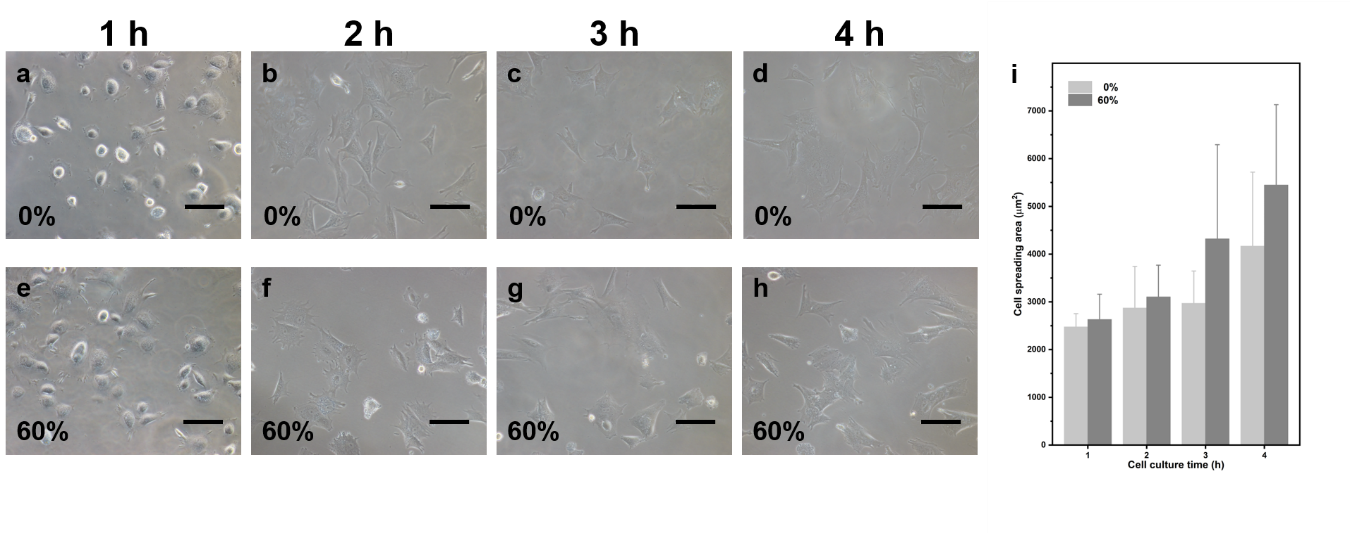


**Figure S1. Culture diagrams of cells at different times on substrates with elongations of 0% and 60%**. The experiment used 14% (w/v) gelatin microfiber with an amplitude of 2 mm and a period of 2 mm to modify 8% (w/v) gelatin matrix. As the culture time prolonged, the spreading area of the cells (mesenchymal stem cells) on the substrate with different elongation gradually increased (**a-h**), and the spreading area was larger on the substrate with elongation 60% (**i**). Scale bars: 100 um.
